# Decoding Unattended Fearful Faces with Whole-Brain Correlations: An Approach to Identify Condition-Dependent Large-Scale Functional Connectivity

**DOI:** 10.1371/journal.pcbi.1002441

**Published:** 2012-03-29

**Authors:** Spiro P. Pantazatos, Ardesheer Talati, Paul Pavlidis, Joy Hirsch

**Affiliations:** 1 fMRI Research Center, Columbia University, New York, New York, United States of America; 2 Department of Physiology and Cellular Biophysics, Columbia University, New York, New York, United States of America; 3 Department of Psychiatry, Columbia University, New York, New York, United States of America; 4 Department of Psychiatry, University of British Columbia, Vancouver, British Columbia, Canada; 5 Centre for High-throughout Biology, University of British Columbia, Vancouver, British Columbia, Canada; 6 Department of Neuroscience, Columbia University, New York, New York, United States of America; 7 Department of Radiology, Columbia University, New York, New York, United States of America; 8 Department of Psychology, Columbia University, New York, New York, United States of America; Indiana University, United States of America

## Abstract

Processing of unattended threat-related stimuli, such as fearful faces, has been previously examined using group functional magnetic resonance (fMRI) approaches. However, the identification of features of brain activity containing sufficient information to decode, or “brain-read”, unattended (implicit) fear perception remains an active research goal. Here we test the hypothesis that patterns of large-scale functional connectivity (FC) decode the emotional expression of implicitly perceived faces within single individuals using training data from separate subjects. fMRI and a blocked design were used to acquire BOLD signals during implicit (task-unrelated) presentation of fearful and neutral faces. A pattern classifier (linear kernel Support Vector Machine, or SVM) with linear filter feature selection used pair-wise FC as features to predict the emotional expression of implicitly presented faces. We plotted classification accuracy vs. number of top N selected features and observed that significantly higher than chance accuracies (between 90–100%) were achieved with 15–40 features. During fearful face presentation, the most informative and positively modulated FC was between angular gyrus and hippocampus, while the greatest overall contributing region was the thalamus, with positively modulated connections to bilateral middle temporal gyrus and insula. Other FCs that predicted fear included superior-occipital and parietal regions, cerebellum and prefrontal cortex. By comparison, patterns of spatial *activity* (as opposed to *interactivity*) were relatively uninformative in decoding implicit fear. These findings indicate that whole-brain patterns of interactivity are a sensitive and informative signature of unattended fearful emotion processing. At the same time, we demonstrate and propose a sensitive and exploratory approach for the identification of large-scale, condition-dependent FC. In contrast to model-based, group approaches, the current approach does not discount the multivariate, joint responses of multiple functional connections and is not hampered by signal loss and the need for multiple comparisons correction.

## Author Summary

Brain activity is increasingly characterized by patterns of pair-wise correlations (large-scale functional connectivity) across the whole brain obtained from Blood Oxygen Level Dependent (BOLD) functional magnetic resonance imaging (fMRI). Typically this is done during resting states (i.e. no presented stimulus) to differentiate subjects based on individual variation or diagnosis. In the current work, we identify such patterns that are a sensitive signature of unattended processing of threat-related stimuli, allowing one to “brain-read” whether an individual was presented with a neutral or fearful face while they attended to non-expression-related stimulus features. These results further the understanding of the neural mechanisms sub-serving threat-detection and facial affect processing in healthy subjects, and may also help further our understanding of various disorders, such as anxiety and autism, which exhibit anomalies in these processes. At the same time, we propose an exploratory and sensitive approach for the identification of condition-dependent, large-scale functional connectivity. This approach is not based on statistical inference on functional connections averaged across subjects and contrasted between two conditions, but rather based on the informative contribution of each functional connection when attempting to predict between two conditions, using machine-learning based multivariate pattern analysis on training data from separate subjects.

## Introduction

Faces with a fearful expression are thought to signal the presence of a significant, yet undetermined source of danger within the environment, or ‘ambiguous threat’ [1]. Evidence from fMRI and evoked potentials (ERPs) suggest that fearful face processing can strongly affect brain systems responsible for face recognition and memory during implicit (consciously perceived but unattended) presentation of these stimuli [2], [3]. Group-based fMRI studies have shown that the perception and processing of facial emotional expression engages multiple brain regions including the fusiform gyrus, superior temporal sulcus, thalamus, as well as affect-processing regions such as amygdala, insula, anterior cingulate cortex among others [4]–[7]. However, to the authors' knowledge, no study to date has identified features of brain activity that contain sufficient information to reliably decode, or “brain-read”, the threat-related emotional expression of unattended (implicitly perceived) faces within individual subjects. The identification of such features, though less well quantified as in group model-based studies, would have a greater capacity for representing distinctions between different cognitive-emotional perceptual states [8], and hence could contribute in advancing our understanding of the neural mechanisms that underlie threat detection and facial emotion processing.

Most group fMRI approaches that have studied the neural correlates of emotional face perception have relied on univariate approaches [9]–[11] which identify regions correlated with a regressor-of-interest, but ignores any interactions with other regions. Bivariate approaches have been applied, but assess the interactivity (functional connectivity) of only one seed region (usually amygdala) with the rest of the brain [12], [13]. Even though several notable studies have taken a multivariate approach in assessing the effective connectivity among multiple brain regions during emotional face processing [14]–[16], a limited number of nodes were included in the networks and they were selected based on a priori anatomical knowledge or on their activation in conventional, General Linear Model (GLM)-based mass univariate analyses. However, univariate GLM approaches make strong assumptions about the hemodynamic response (i.e. sustained periods of activation or deactivation relative to baseline), while functional connectivity offers a complementary and more data-driven and exploratory measure that makes use of temporal correlations to estimate functional connectivity [17].

There has been a recent surge of interest in examining the large-scale (i.e. pair-wise connectivity throughout the whole-brain) functional network architecture of the brain as a function of various cognitive processes or individual variation [18]. This is often done by first defining a set of functional “nodes” based on spatial ROIs and then conducting a connectivity analysis between the nodes based on their FMRI timeseries. Large-scale functional connectivity patterns have been successful in predicting age [19] as well as subject-driven mental states such as memory retrieval, silent-singing vs. mental arithmetic [20] and watching movies vs. rest [21]. It remains to be determined however, whether whole-brain connectivity can be used to decode very similar stimuli that differ by only one or a few subtle characteristics, such as the emotional expression of an unattended face. If so, then functional connections that discriminate between the two conditions can be interpreted as being uniquely related to the parameter of interest that varies across both conditions.

Although multivariate pattern analyses are more sensitive than group, model-based approaches, one disadvantage is decreased interpretability and quantification of the precise relationship among features related to a certain condition [8]. However, since this approach exploits the information inherent in the joint responses of many functional connections, an advantage is that pattern classification of similar conditions coupled with feature selection and identification can be used as a means to identify condition-dependent, large-scale functional connectivity, without the need to correct for tens of thousands of multiple comparisons. This approach can be used for hypothesis generation to identify groups of functional connections associated with a condition, which can then serve as connections and regions of interest for more rigorous and mechanistically revealing approaches such as effective connectivity [22].

**Figure 1:**
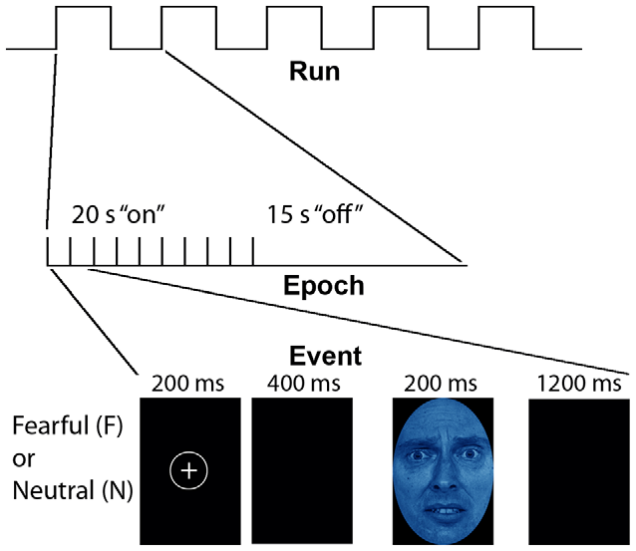
Experimental paradigm for the interaction of attention and affect (adapted from Etkin, et. al. 2004). Stimuli were either fearful (F) or neutral (N) expression faces, pseudocolored in red, yellow,or blue. Each event was comprised of a face which was either masked (33 ms for a fearful or neutral face, followed by 167 ms of a neutral face mask of the same gender and color, but different individual; MF or MN, respectively), or unmasked (200 ms for each face; F or N) or masked. Ten events of the same type, spaced 2 seconds apart, were presented within each 20 second block, followed by 15 seconds of crosshair with black background. There were four blocks per condition, giving 40 time points in the correlation estimates per condition per subject. In view of our specific hypotheses, only the unmasked conditions are discussed in the main text, while results for unmasked conditions are presented elsewhere (manuscript in preparation).

**Figure 2:**
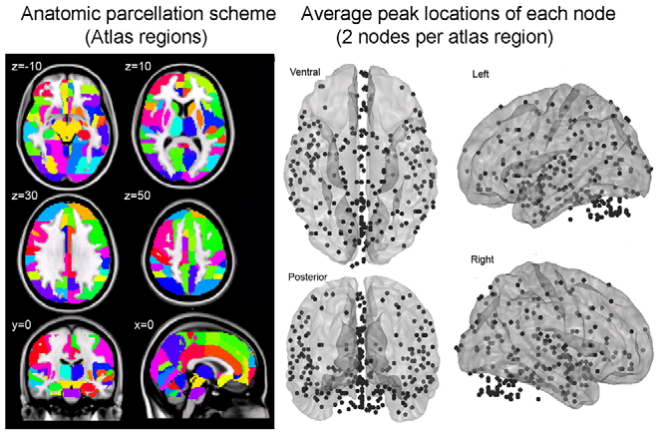
Node definitions and anatomical locations. Cortical and subcortical regions (ROIs) were parcellated according to bilateralized versions of the Harvard-Oxford Cortical and subcortical-atlases, and the cerebellum was parcellated according to AAL (left panel). ROIs were trimmed to ensure there was no overlap between them and that they contained voxels present in each subject. The top two eigenvariates from each ROI was extracted, resulting in 270 total nodes throughout the brain (right panel). For display purposes, node locations (black spheres) correspond to the peak loading value from each time-course's associated eigenmap averaged over all subjects.

Here we estimate the large-scale functional networks of implicit fear processing using a blocked design and Blood Oxygen Level Dependent (BOLD) image acquisition, during which subjects were instructed to identify the color of pseudo-colored fearful and neutral faces (Figure 1). We applied atlas-based parcellation to derive several hundred nodes throughout the whole-brain and computed thousands of pair-wise correlations (40 total time points, or 80 s worth of fMRI data) during each of two conditions: implicit processing of fearful and neutral faces. We then employed multivariate pattern analyses in conjunction with linear filter feature selection to identify functional connections whose pattern could distinguish between implicit processing of fearful and neutral faces within individual subjects, using training data from separate subjects. We plotted classification accuracy vs. number of included features to approximate the minimum number of informative features, and then identified these features (functional connections) on a neuroanatomical display. See Figure 2 for an outline of the analysis scheme.

Our primary objective was to test the hypothesis that condition-specific, functional connectivity over the whole-brain (here Pearson correlation using 40 time points of fMRI data per example) contain enough information to discriminate between implicitly presented fearful and neutral faces, and to identify the functional connections that are most informative in this decoding task. A secondary objective was to compare the decoding accuracies achieved when using *interactivity* (pair-wise correlations) vs. *activity* (i.e. beta estimates from SPM maps). We show that a small subset of connections estimated across the whole-brain can predict, or “brain-read”, implicitly presented fearful faces with high peak accuracies using training and testing data from separate subjects. We propose that this is a valuable, exploratory approach to estimate condition-dependent, large-scale functional connectivity and demonstrate that whole-brain patterns of *interactivity* are a sensitive and informative signature of cognitive-emotional perceptual states.

## Materials and Methods

### Ethics statement

All procedures and tasks were reviewed for ethical concerns and protection of human subjects by appropriate local IRB boards prior to subject recruitment and data collection. The procedures described in this study of healthy adults have been approved by the Columbia University Morningside IRB (#IRB-AAAA3690, PI: Joy Hirsch) and IRB (#IRB5290, PI: Myrna M. Weissman)

### Subjects

A total of 38 (19 female) healthy volunteers (mean age = 29, SD = 6.9) with emmetropic or corrected-to-emmetropic vision participated in the study in accordance with institutional guidelines for research with human subjects. All subjects were screened to be free of severe psychopathology including Bipolar Disorder and Psychotic Disorders.

### Stimulus presentation paradigm

Subjects performed a previously described task (Etkin, Klemenhagen et al. 2004) which consists of color identification of fearful and neutral faces (F and N respectively). Although backwardly masked (subliminal) fearful and neutral faces were also presented, here we discuss results based on the unmasked (supraliminal) conditions. Results based on comparisons of masked conditions are presented elsewhere (manuscript in preparation). *Stimuli:* Black and white pictures of male and female faces showing fearful and neutral facial expressions were chosen from a standardized series developed by Ekman and Friesen [23]. Faces were cropped into an elliptical shape that eliminated background, hair, and jewelry cues and were oriented to maximize inter-stimulus alignment of eyes and mouths. Faces were then artificially colorized (red, yellow, or blue) and equalized for luminosity. For the training task, only neutral expression faces were used from an unrelated set available in the lab. These faces were also cropped and colorized as above.

### Behavioral task

Each stimulus presentation involves a rapid (200 ms) fixation to cue subjects to fixate at the center of the screen, followed by a 400 ms blank screen and 200 ms of face presentation. Subjects have 1200 ms to respond with a key press indicating the color of the face. Behavioral responses and reaction times were recorded. Unmasked stimuli consist of 200 ms of a fearful or neutral expression face, while backwardly masked stimuli consist of 33 ms of a fearful or neutral face, followed by 167 ms of a neutral face mask belonging to a different individual, but of the same color and gender (see Figure 1). Each epoch consists of ten trials of the same stimulus type, but randomized with respect to gender and color. The functional run has 16 epochs (four for each stimulus type) that are randomized for stimulus type. To avoid stimulus order effects, we used two different counterbalanced run orders. Stimuli were presented using Presentation software (Neurobehavioral Systems, http://www.neurobs.com), and were triggered by the first radio frequency pulse for the functional run. The stimuli were displayed on VisuaStim XGA LCD screen goggles (Resonance Technology, Northridge, CA). The screen resolution was 800×600, with a refresh rate of 60 Hz. Prior to the functional run, subjects were trained in the color identification task using unrelated neutral face stimuli that were cropped, colorized, and presented in the same manner as the nonmasked neutral faces described above in order to avoid any learning effects during the functional run. After the functional run, subjects were shown all of the stimuli again, alerted to the presence of fearful faces, and asked to indicate whether they had seen fearful faces on masked epochs.

### fMRI acquisition

Functional data were acquired on a 1.5 Tesla GE Signa MRI scanner, using a gradient-echo, T2^*^-weighted echoplanar imaging (EPI) with blood oxygen level-dependent (BOLD) contrast pulse sequence. Twenty-four contiguous axial slices were acquired along the AC-PC plane, with a 64×64 matrix and 20 cm field of view (voxel size 3.125×3.125×4 mm, TR = 2000, TE = 40, flip angle = 60). Structural data were acquired using a 3D T1-weighted spoiled gradient recalled (SPGR) pulse sequence with isomorphic voxels (1×1×mm) in a 24 cm field of view (256×256 matrix, ∼186 slices, TR 34 ms, TE 3 ms).

### GLM analysis

Functional data were preprocessed and processed in SPM8 (Wellcome Department of Imaging Neuroscience, London, UK). For preprocessing, the realigned T2*-weighted volumes were slice-time corrected, spatially transformed and resampled to a standardized brain (Montreal Neurologic Institute, 2×2×2 mm^3^ cube resolution) and smoothed with a 8-mm full-width half-maximum Gaussian kernel. 1st-level regressors were created by convolving the onset of each block (MF, MN, F and N) with the canonical HRF with duration of 20 seconds. Additional nuisance regressors included 6 motion parameters, white matter and csf signal, which were removed prior to time-series extraction. For the current work, the same GLM analysis served three purposes: 1) facilitate removal of nuisance effects from time series prior to FC estimation using structurally (atlas-based) and functionally defined ROIs, 2) produce beta-estimates of each condition for classification analysis of spatial activity patterns and 3) functionally define ROIs (nodes) prior to FC calculation (used for comparing results of structural vs. functional definition of nodes).

### Node definitions

**Figure 3:**
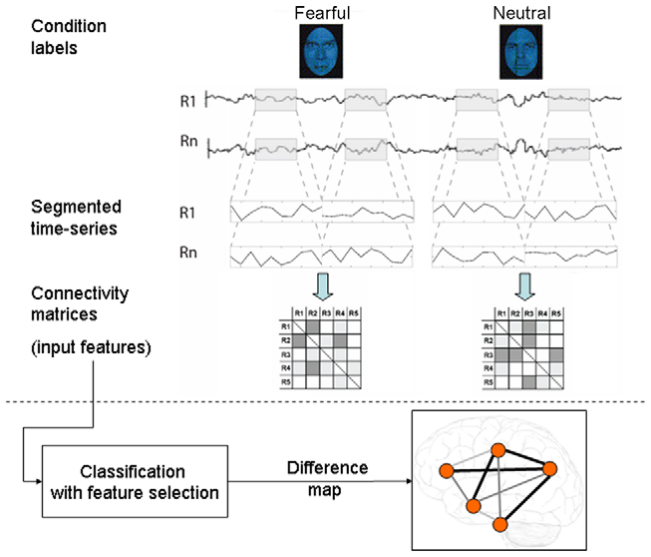
Data analysis scheme. Time series from each condition (unmasked fearful and unmasked neutral, F and N) and for N regions (R1 though RN) were segmented from each subject's whole run and concatenated (concatenation of two blocks for each condition shown in figure). There were four 20 second (10 TR) blocks of each condition; hence each example was comprised of 40 time points per condition per subject. For each of example, correlation matrices were estimated, in which each off-diagonal element contains Pearson's correlation coefficient between region *i* and region *j*. The lower ***triangular*** region of each of these matrices were used as input features in subsequent classifiers that learned to predict the example (i.e. F or N) based on their observed patterns of the correlations. Here, we used a filter feature selection based on t-scores in the training sets during each iteration of leave-two-out cross validation. The difference map consists of the set of most informative features (those that are included in the most rounds of cross-validation and have the highest SVM weights.)

Brain regions were parcellated according to bilateral versions of the Harvard-Oxford Cortical and sub-cortical atlases and the AAL atlas (cerebellum) and were trimmed to ensure no overlap with each other and to ensure inclusion of only voxels shared by all subjects (Figure 3, left panel). For each subject, time-series across the whole run (283 TRs) were extracted using Singular Value Decomposition (SVD) and custom modifications to the Volumes-of-Interest (VOI) code within SPM8 to retain the top 2 eigenvariates from each atlas-based region. Briefly, the data matrix for each atlas-based region is defined as A, an n×p matrix, in which the n rows represent the time points, and each p column represents a voxel within an atlas-based region. The SVD theorem states:
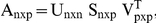
where U^T^U = I_nxn_ and V^T^V = I_pxp_ (i.e. U and V are orthogonal). The columns of U are the left singular vectors (eigenvariates, or summary time courses of the region), S (the same dimensions as A) has singular values, arranged in descending order, that are proportional to total variance of data matrix explained by its corresponding eigenvariate, and is diagonal, and V^T^ has rows that are the right singular vectors (spatial eigenmaps, representing the loading of each voxel onto its corresponding eigenvariate). Here we retain the top two eigenvariates (nodes) from each region.

For each atlas-based region, we opted to apply SVD over the entire time-series from each subject and *then* segment and concatenate the eigenvariates according to the conditions/comparisons of interest (rather than segment and concatenate all the masks' voxels *first* and then apply SVD) in order to maximize the total number of observations (time points) per region and also to avoid potentially introducing any artifact and unnatural variation caused by the splicing together of signal from disparate time points, which could possibly bias the SVD results. However, a potential disadvantage of this approach is that important sub-regions and associated eigenvariates within a particular atlas-based region could be missed due to variation in other conditions/blocks within the run that are not considered in the current work. This is an additional motivation to retain the top two eigenvariates from each atlas-based region, as opposed to just one.

The above step resulted in a total of 270 nodes with an associated time course (i.e. eigenvariates) and spatial eigenmaps from the 135 initial atlas-based regions. Thus, each atlas-based region was comprised of two nodes. Interestingly, when extracting only one eigenvariates per region, maximum accuracy did not surpass 46% (data not shown). This is possibly due to the fact that larger, atlas-based regions encompassed other functional sub-regions which were not included in the analysis. Another possible reason is that for many regions, the 1^st^ eigenvariate may reflect artifact global or mean grey matter signal (while white matter and csf signal were regressed out from nodes' time-series, global and mean grey matter signals were not), or it may reflect variation caused by other conditions/blocks within the run that were not considered in the current classification analyses (see paradigm task description above), or a combination of all the above. Therefore we extracted two eigenvariates from each region. We note that this means it is likely that node 2 of a particular region shows functional connectivity that differentiates between conditions and node 1 of the same region has no differential connectivity. For clarity we therefore label each node using its Harvard-Oxford atlas label appended by either “_PC1” for the first eigenvariate and “_PC2” for the second. For display purposes, we calculated the MNI coordinates of the peak loading weight (locations averaged across subjects) for each eigenvariate from its associated eigenmap (Figure 3, right panel). Table S1 lists these average MNI coordinates for each node.

### Functional connectivity networks for implicit fearful and neutral face processing

For each subject, functional connectivity matrices (i.e. where cell *i,j* contains the Pearson correlation between region *i* and region *j*) were generated for implicit fearful (F) and neutral (N) conditions. The above time-series were segmented and concatenated according to conditions of interest (40 total time points per condition, incorporating a lag of 2 or 3 s from the start of each block) before generating the correlation matrices. Fisher's R to Z transform was then applied to each correlation matrix. Finally for the binary classification of interest (i.e. F vs. N), correlation matrices were demeaned with respect to the average between the two conditions in order to remove the effects of inter-subject variability. The lower diagonal of the above preprocessed correlation matrices (38 subjects×2 conditions total) were then used as input features to predict viewed stimuli in subsequent pattern recognition experiments.

### Differences in functional connectivity between implicit fearful and neutral face processing

We first tested for significant differences between the primary conditions of interest (i.e. F>N) while correcting for multiple comparisons (False Discovery Rate, FDR). This yielded no significant results when multiple comparison correction was applied (FDR, p<0.05 and 0.1). This was not surprising, as multiple comparison correction was expected to be too conservative given the exceedingly high number of independent comparisons (36,315).

### Pattern analysis of large-scale functional connectivity to predict implicit fear perception

Support vector machines are pattern recognition methods that find functions of the data that facilitate classification [24]. During the training phase, an SVM finds the hyperplane that separates the examples in the input space according to a class label. The SVM classifier is trained by providing examples of the form <*x*,*c*>, where x represents a spatial pattern and *c* is the class label. In particular, x represents the fMRI data (pattern of correlation strengths) and *c* is the condition or group label (i.e. *c* = 1 for F and *c* = −1 for N). Once the decision function is determined from the training data, it can be used to predict the class label of new test examples.

For all binary classification tasks, we applied a linear kernel support vector machine (SVM) with a filtering feature selection based on t-test and leave-two-out cross validation (LTOCV). There were 38 examples for each condition (2 from each subject, 76 total). During each iteration of 38 rounds of LTOCV, both examples (1 from each class) from one subject were withheld from the dataset and 1) a 2-sample t-test was performed over the remaining training data (N = 37 in each group) 2) the features were ranked by absolute t-score and the top N were selected 3) these selected features were then used to predict the class of the withheld test examples during the classification stage. The full feature set for each example consisted of 36,315 correlations.

If the classifier predicted all trials as positive or negative, the resulting accuracy would be 50% since the number of examples are equal for each class. We therefore report classification accuracy (number of true positives and negatives over all trials) vs. number of included features that have been ranked by their t-score. We assessed the significance of decoding results by computing the frequency in which actual values surpassed those from null distributions derived by randomly permuting class labels based on the method proposed by [25], with the a slight modification to account for the dependence between pairs of examples from each subject. Briefly, to derive this null distribution, class labels within each pair conditions from each subject were randomly flipped with a probability of 0.5 over 2000 iterations for each number of included features. P-values for the peak decoding accuracies (F vs. N: 100%, top 25 features) were also calculated with respect to classification results when shuffling labels 10,000 times, and then subjected to Bonferroni correction for the number of total Top N comparisons (in this case 20).

For SVM learning and classification we used the Spider v1.71 Matlab toolbox (http://people.kyb.tuebingen.mpg.de/spider/) using all default parameters (i.e. linear kernel SVM, regularization parameter C = 1. Graphical neuro-anatomical connectivity maps of the top N features were displayed using Caret v5.61 software (http://brainvis.wustl.edu/wiki/index.php/Caret:About). We note that different features could be selected during the feature selection phase of each round of cross-validation. Therefore in ranking the top 25 features, we first rank by total number of times that feature was included in each round of cross-validation, and then among these features, we sort by absolute value of the average SVM weight.

Our intent is not to estimate the true accuracy of prediction given a completely new data set, but rather to test whether there exists information in the pattern of functional connections relevant to unattended emotion perception, and to approximate the optimal number of features that containing this information. We note that our approach (plotting accuracy vs. number of top N features) is not biased, since for each number of top N features, and for each round of leave-two-out cross validation, the top N features were selected from a training set that was completely independent from the testing set. If there is a true signal present in the data, we expect, and in the current data in general observe, that there is an initial rise in accuracy as more informative features are added to the feature set, and a dip in accuracy as less informative features (i.e. noise) are added to the feature set. Therefore in reporting classification results, we report the range of features at which accuracies first reach maximum accuracy-10% (positive slope) to which they reach maximum accuracy-10% (negative slope), and also correct for multiple comparisons (i.e. number of top N features tested) using Bonferroni when reporting the p-value for the maximum accuracy achieved.

For assessing the significance of the differences between decoding results (i.e. FC as features vs. beta estimates) we used the Accurate Confidence Intervals MATLAB toolbox for assessing whether the parameter *p* (probability of correct prediction) from two independent binomial distributions was significantly different (http://www.mathworks.com/matlabcentral/fileexchange/3031-accurate-confidence-intervals). Briefly, these methods search for confidence intervals using an integration of the Bayesian posterior with diffuse priors to measure the confidence level of the difference between two proportions [26]. We used the code prop–diff(*x*_1_,*n*_1_,*x*_2_,*n*_2_,delta), (available from the above website) returning Pr(*p*_1_−*p*_2_>*δ*), where *x*_1_, *n*_1_, *x*_2_, *n*_2_, are number of correct responses and total predictions in two distributions being compared, and delta (zero in our case) is the null hypothesis difference between the probabilities.

## Results

### Behavioral results

The average response rate in the color discrimination task was 98% (σ = 4.6%), mean accuracy was 97% (σ = 3.5%), and mean reaction time was 0.65 s (σ = 0.12), indicating that subjects performed the color discrimination task as instructed.

### Discriminating between implicit processing of fearful and neutral faces with patterns of functional connectivity

We applied atlas-based parcellation (see Figure 2) and computed pair-wise correlations between 270 nodes (derived from 135 atlas-based brain regions) using 40 total time points of fMRI data that were segmented and concatenated from two conditions; unattended and nonmasked (i.e. implicit) fearful (F) and neutral (N) faces (Figure 1). This resulted in 36,315 total functional connections (z-transformed Pearson correlations) for each condition of interest (F and N).

We quantified the extent to which a subset of these functional connections could decode, or predict, the conditions from which they were derived by submitting them as features into a pattern classifier. We used a linear kernel Support Vector Machine (SVM) with a filter feature selection based on the t-score of each feature (functional connectivity) in each training set. Decoding accuracies for implicit fearful vs. neutral classifications (F vs. N) were plotted against the number of included features (ranked in descending order by t-score) in order to approximate the number of informative features relevant to the emotional expression of the facial stimulus.

**Figure 4:**
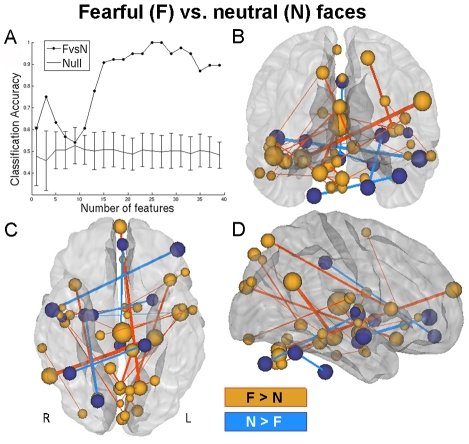
Large-scale functional connectivity discriminates between unattended, conscious processing of fearful and neutral faces. (**A**) Decoding accuracy when classifying F vs. N as a function of the number of features (1 to 40) included ranked in descending order by their absolute t-score. Maximum accuracy for F vs. N classification (100%, p<0.002, corrected) was achieved when learning was based on the top 25 features in each training set. Mean accuracy scores for shuffled data are plotted along the bottom, with error bars representing standard deviation about the mean. Posterior (**B**), ventral (**C**) and right lateralized (**D**) anatomical representation of the top 25 features when classifying supraliminal fearful vs. supraliminal neutral face conditions (F vs. N). The thalamus (large red sphere in the center of each view) is the largest contributor of connections the differentiate the F from N. Red indicates correlations that are greater in F, and blue represents correlations that are greater in N. For display purposes, the size of each sphere is scaled according to the sum of the SVM weights of each node's connections, while the color of each sphere is set according to the sign of this value; positive sign, red, F>N and negative sign, blue, N>F. In addition, the thickness of each connection was made proportional to its SVM weight.

For implicit fearful vs. neutral (F vs. N) classification, accuracy reached 90% when learning was based on the top 15 features in each training set, a maximum of 100% (p<0.002, corrected) at 25 features, and dipped back down to 90% at about 35 features (Figure 4A). Anatomical display of the top 25 overall features that differed between F and N conditions revealed functional connections among occipital regions, middle and superior temporal gyrus, lateral and medial prefrontal regions, thalamus, cerebellum and insula (Figure 4B–D, Table 1). The connection that carried the most weight in the linear SVM classifier was between right angular gyrus and left hippocampus, which exhibited a greater correlation in the F vs. N condition (Table 1, F# 1). To identify regions whose overall functional connectivity was greater during fear, the size of each node was made proportional to the sum of SVM weights of each of its connections. The node with the most positive functional connectivity during fear was the thalamus (Figure 4B–D, large red sphere in center), which exhibited positively modulated functional connections with bilateral middle temporal gyrus and right insula.

**Table 1:** F vs. N, Top 25 features (consensus features are in bold).

F#	Edge label	Mean R (F)	Mean R (N)	T-value	SVM weight	FSets
**1**	**Right_Angular_Gyrus_PC1 - Left_Hippocampus_PC2**	**0.101**	**−0.027**	**4.3419**	**1.1347**	**38**
**2**	**Right_Superior_Temporal_Gyrus_anterior_division_PC2 - Left_Ventral_Frontal_Pole_PC1**	**−0.08**	**0.066**	**−4.301**	**−0.9976**	**38**
**3**	**Right_Dorsal_Frontal_Pole_PC2 - Cerebelum_6_L_PC2**	**0.07**	**−0.092**	**4.3555**	**0.97075**	**38**
**4**	**Vermis_7_PC2 - Midbrain_PC1**	**0.127**	**7E-04**	**4.2176**	**0.88976**	**38**
**5**	**Right_Temporal_Occipital_Fusiform_Cortex_PC2 - Pons_PC2**	**−0.07**	**0.082**	**−4.4395**	**−0.8891**	**38**
**6**	**Right_Putamen_PC2 - Cerebelum_Crus1_R_PC2**	**−0.07**	**0.094**	**−5.5049**	**−0.8803**	**38**
**7**	**Left_Frontal_Orbital_Cortex_PC2 - Left_Cuneal_Cortex_PC2**	**0.052**	**−0.082**	**4.4034**	**0.84121**	**38**
**8**	**Right_Frontal_Operculum_Cortex_PC2 - Right_Dorsal_Lateral_Occipital_Cortex_superior_division_PC2**	**0.118**	**−0.027**	**5.5009**	**0.81892**	**38**
9	Right_Frontal_Medial_Cortex_PC1 - Right_Cingulate_Gyrus_posterior_division_PC2	0.003	0.133	−3.943	−0.8083	19
10	Right_Amygdala_PC2 - Left_Putamen_PC1	0.009	0.131	−4.1008	−0.7664	34
**11**	**Right_Lingual_Gyrus_PC1 - Left_Dorsal_Lateral_Occipital_Cortex_superior_division_PC2**	**0.088**	**−0.068**	**4.1602**	**0.7472**	**38**
**12**	**Left_Thalamus_PC2 - Left_Planum_Polare_PC1**	**0.091**	**−0.076**	**4.7585**	**0.65859**	**38**
**13**	**Left_Temporal_Occipital_Fusiform_Cortex_PC2 - Cerebelum_8_L_PC1**	**0.043**	**−0.102**	**4.3388**	**0.62211**	**38**
**14**	**Right_Central_Opercular_Cortex_PC2 - Left_Lingual_Gyrus_PC2**	**0.061**	**−0.077**	**4.3741**	**0.61316**	**38**
15	Vermis_8_PC1 - Left_Planum_Polare_PC2	0.085	−0.042	3.9352	0.59068	19
16	Right_Insular_Cortex_PC2 - Left_Caudate_PC2	0.028	−0.089	3.873	0.57516	11
**17**	**Right_Parahippocampal_Gyrus_anterior_division_PC1 - Left_Middle_Temporal_Gyrus_anterior_division_PC2**	**−0.02**	**−0.151**	**4.1911**	**0.55492**	**38**
18	Right_Ventral_Lateral_Occipital_Cortex_superior_division_PC2 - Right_Middle_Temporal_Gyrus_posterior_division_PC2	0.011	−0.074	3.8763	0.55272	15
**19**	**Right_Central_Opercular_Cortex_PC1 - Left_Planum_Polare_PC1**	**0.077**	**0.219**	**−4.2479**	**−0.5409**	**38**
20	Left_Juxtapositional_Lobule_Cortex_Supp_Motor_cortex_PC2 - Left_Inferior_Frontal_Gyrus_pars_triangularis_PC2	0.041	−0.073	3.9504	0.48896	20
21	Right_Precuneous_Cortex_PC1 - Left_Middle_Temporal_Gyrus_anterior_division_PC1	−0.01	−0.12	3.8799	0.43938	15
**22**	**Left_Thalamus_PC2 - Left_Insular_Cortex_PC1**	**0.085**	**−0.057**	**4.2959**	**0.42672**	**38**
23	Right_Planum_Polare_PC2 - Cerebelum_Crus2_L_PC2	0.043	−0.083	3.8435	0.41322	12
**24**	**Right_Planum_Polare_PC1 - Left_Thalamus_PC2**	**0.068**	**−0.093**	**4.1779**	**0.39581**	**38**
25	Left_Cingulate_Gyrus_anterior_division_PC1 - Hypothalamus_PC2	0.049	−0.059	3.8567	0.38869	13

In addition to parcelating the brain and defined nodes based on an atlas, we also functionally defined nodes using two approaches 1) using the same 160 MNI coordinates as used in Dosenbach et. al., 2010 [19] which were selected and defined based on separate meta-analyses of the fMRI literature, and 2) a biased approach based on 92 nodes (2 eigenvariates from each of 49 ROIs defined as 6 mm radius spheres centered at peak coordinates) that were based on the GLM results from the same, whole dataset (for F contrast F>N thresholded at p = 0.05, k = 30). For 1) achieved accuracies were 63–73% when using 75 to 130 features, and for 2) accuracies between 76–86% were obtained when using 80 to 140 features (data not shown). Approach 2) is biased in that we defined our nodes based on the GLM results of the whole data set, and as such provides an upper bound on the expected accuracies when functionally defining nodes based on the GLM results in separate training sets during each iteration of LTOCV. Therefore we conclude that the above whole-brain, atlas-based approach, which achieved 90–100% accuracy with 15–35 features when using unbiased feature selection, is optimal to using functionally defined nodes.

### Discriminating between F and N faces using spatial patterns of activation

**Figure 5:**
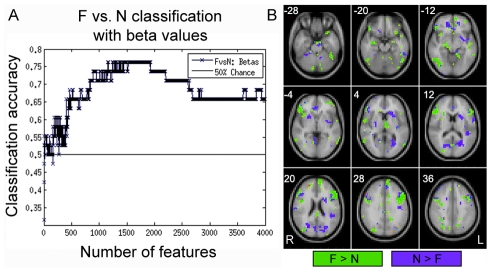
Classification results using beta estimates as features. (**A**) Feature selection, cross-validation and SVM learning were performed exactly the same as for FC, but over the range of 1 to 4000 ranked features (voxels). Accuracies for F vs. N classification reached 66–76% with ∼500–2500 features, with maximum accuracy (76%, p = 0.0044, uncorrected) at ∼1,900 features. (**B**) The most informative voxels with positive SVM weights (F>N, yellow) included fusiform gyrus (−28,−20,−12), cerebellum (−28, −20), amygdala (−20), insula (−12), orbital and ventrolateral prefrontal cortex (−20, −12, −4), midbrain (−12), parahippocampal gyrus (−12), middle temporal gyrus and superior temporal sulcus (−12,−4,4), thalamus/pulvinar (4), dorsolateral prefrontal/opercular cortex (12,20,28), dorsomedial prefrontal cortex (20,28), and superior occipital cortex (20,28) and inferior parietal lobe (36). Informative voxels with negative SVM weights (N>F, blue) included temporal-occipital cortex (−20), subgenual anterior cingulate (−12,−4), striatum (−4,4), lingual gyrus (4,12), precuneus (20) and dorsolateral prefrontal cortex (28,36). (B). Brain images are displayed using Neurological convention (i.e. L = R), and top left number in each panel represents the MNI coordinate (z) of depicted axial slice.

To compare the information content of patterns of *interactivity* (i.e. functional connections used above) vs. patterns of *activity* we also attempted F vs. N classifications using beta estimates, which are considered summary measures of activation in response to each condition. In order to make feature-selection/LTOCV and SVM learning more computationally tractable, preprocessed functional data were resized from 2×2×2 mm voxel resolution to 4×4×4 mm resolution, and subject-specific GLM models were re-estimated, resulting in a reduction of total feature space per example from ∼189,500 betas to ∼23,500. Feature selection, LTOCV and SVM learning proceeded exactly as above for FC data. We observed accuracies of 66%–76% with ∼500 to 2600 features, with peak accuracy at 76% (p = 0.0044, uncorrected) at ∼1900 features (Figure 5A). The most informative voxels encompassed many distributed regions that included dorsolateral prefrontal/opercular cortex, fusiform gyrus, lateral occipital cortex, superior temporal gyrus, anterior cingulate, amygdala, parahippocampal gyrus, ventrolateral prefrontal cortex, pulvinar, precuneus, cerebellum, inferior parietal lobe and insula (Figure 5B). Although significantly above chance, and despite the involvement of many more regions, maximum accuracy using betas was significantly less than the maximum accuracy achieved with FC (76%<100%, p = 5.37×10−7).

We performed additional classifications using betas derived from the original, smaller voxel-sizes and with the addition of an initial (positively biased) feature selection step over the whole-dataset for the same issues of technicality stated above. This also served to estimate an upper bound on the expected accuracy when using beta-values: if maximum accuracy achieved was still less than when using functional connectivity with unbiased feature selection, then we can more readily conclude that functional connectivity features are more “informative” than beta estimates (when using the Canonical Hemodynamic Response Function (HRF) to model activation). For this analysis, the initial (biased) feature selection employed an F-test of the contrast F>N thresholded at p<0.01, cluster threshold = 20, resulting in 4,226 total initial features. Feature selection/LTOCV and classification again proceeded as above across the range of 1 to 4000 features. In spite of initially biased feature selection, F vs. N classification reached 92% maximum accuracy (data not shown).

In addition to using beta maps throughout the whole-brain, we derived beta weights using the same summary time courses (eigenvariates) that were extracted and used to compute pair-wise FC (270 total betas per condition per subject). For this, the GLM analysis was kept the same as above except that previously included nuisance regressors (6 motion, mean white and mean csf) and a low-pass filter were not included, since they were already removed from the time courses during extraction. Resulting estimated beta weights were then used as features to predict fearful vs. neutral faces using the exact same procedure when using whole-brain FC. Accuracies of between 69–79% were achieved with between 40 to 150 features (data not shown).

## Discussion

Here we demonstrate that pattern analysis of large-scale functional connectivity can reliably decode the emotional expression of implicitly perceived faces, and that pair-wise functional connections are modulated by implicit fear perception. This work also demonstrates a whole-brain, large-scale and exploratory approach for the identification of condition-specific, functional connectivity that avoids correcting for multiple comparisons among thousands of connections (discussed more below).

The most significantly modulated functional connection during implicit presentation of fearful faces was between left hippocampus and right angular gyrus. The left hippocampus is a key region for memory (i.e. autobiographical memory retrieval) and the right angular gyrus has been implicated in mentalizing, or inferring the thoughts and feelings of others [27]. Interestingly, during resting states, these two regions were found not to correlate with each other, but instead correlated with other regions that substantially overlapped, such as superior temporal sulcus (STS), anterior temporal lobe, posterior cingulate cortex, dorsomedial and ventral prefrontal cortex, inferior frontal gyrus, and the amygdala. It has been proposed that this functional overlap facilitates the integration of personal and interpersonal information and provides a means for personal experiences to become social conceptual knowledge [27]. Here, we observed the left hippocampus and right angular gyrus were correlated during implicit emotion (fear) perception, suggesting the integration of autobiographical memory with mentalizing during implicit perception of emotional faces.

Other connections that discriminated between implicitly presented fearful and neutral faces included thalamus, superior occipital, frontal operculum, dorsal-lateral prefrontal cortex, cerebellum, parietal and posterior and anterior temporal regions (in the vicinity of the superior temporal sulcus, STS). This latter observation is consistent with previous models and group studies that identify the STS and middle temporal gyrus as a primary neural substrate for processing the emotional expression of faces [28]–[30], and recent work demonstrating that multivariate pattern analyses applied to these regions could decode explicit emotional face recognition [31]–[33]. Importantly, the current findings suggest that interactions of temporal regions and STS with areas such ventral frontal pole, thamalus, parahippocampal gyrus and central opercular cortex (Table 1 F# 2, 12, 24 and 17) are also critically involved in implicit emotion perception.

Contrary to our expectations, other than a connection between amygdala and putamen (Table 1, F# 10), the top 25 features that discriminated between the implicit fear and neutral conditions did not include any connections with the amygdala. This is not inconsistent with the observation in a recent meta-analysis that amygdala activity was significantly greater for explicit (attended) fear perception vs. implicit fear perception [34]. In addition, the finding that amygdala demonstrates a distinct temporal profile from other structures during emotional face processing could also explain why more functional connections with amygdala were not observed in the current analysis [10]. Instead, the structure which contributed the most in discriminating between the fear and neutral conditions was thalamus (Fig. 4C and D, largest red sphere in center), which exhibited greater correlations with bilateral middle temporal gyrus (STS) and left insula during the fear condition (Table 1 rows 12, 22 and 24). This observation is consistent with its purported role as a hub integrating cortical networks during the evaluation of the biological significance of affective visual stimuli [35], and with the observation of direct structural connectivity between several sub-regions of the thalamus with the STS [36]. The current results suggest that functional connectivity between thalamus and STS and insula play a prominent role during implicit fear perception.

Interestingly, functional connections of the cerebellum were also significantly modulated during the fear condition. In particular, functional connections of the cerebellum with dorsal frontal pole (Table 1 F# 3) and fusiform gyrus (F# 13) were increased during fear, while connections with putamen (F# 6) were decreased. Although cerebellum has been frequently reported to be activated or involved during emotion processing [34], [37], [38], the specific roles the various subregions play during affective processing remain to be elucidated [39].

Previous studies have shown that emotional faces modulate amygdala-fusiform (FG) interactions [14], [40], [41]. Although amygdala-FG interactions did not appear among the top features for discriminating between implicit fearful and neutral faces, we did observe increased amygdala-FG connectivity during implicit fear relative to implicit neutral when we isolated that connection (Right_Temporal_Occipital_Fusiform_Cortex_PC1, MNI = [26,−48,−18] and Right_Amygdala_PC1, MNI = [18,0,−20], t = 2.6, p<0.01), which is consistent with the above works.

### Large-scale functional network of fear processing

It is clear that fearful emotion processing and its behavioral consequences involve the complex interactions among many distributed regions [42]–[44]. Among these, the amygdala and its interactions with the frontal and visual cortex are critically involved in attended and pre-attentive threat and emotion processing [9], [13], [45], [46]. Numerous previous studies have examined functional interactions between amygdala and several other regions in the fear and facial emotion processing pathway. Usually these have used Psycho-Physiological Interaction (PPI) analysis to study the functional connectivity of a seed region, often the amygdala, with the rest of the brain during a fearful relative to non-fear perceptual or cognitive state [12], [46]. Other studies employed effective connectivity measures such as structural equation modeling (SEM) and dynamic casual modeling (DCM) to examine multiple interactions among a more limited set of a priori defined regions [14], [16].

In contrast to the above-mentioned studies, the current approach is relatively model-free in that we estimate functional connectivity throughout the whole-brain without a priori restrictions based on anatomically defined areas or seed regions. We estimate network connections using simple correlation measures, similar to a previous study that demonstrated condition dependent modulations in large-scale (41 nodes) functional connectivity across various syntactical language production tasks [47], but on a much larger scale (270 nodes in the current analysis). We then identified a subset of functional connections whose pattern could discriminate between implicit fearful and neutral face processing.

### An approach to estimate condition specific large-scale functional connectivity

There is considerable interest in examining the large-scale functional network architecture of the brain as a function of various cognitive processes or individual variation [18]. This is often done by first defining a set of functional “nodes” based on spatial ROIs and then conducting a connectivity analysis between the nodes based on their FMRI timeseries. Group-based statistical parametric mapping can then be applied to resulting connections [48]. However, as the number of nodes (N) increases, the number of connections increases exponentially (# connections = (N*(N−1))/2) resulting in a multiple comparisons problem, and hindering the exploration-based query of condition-specific whole-brain functional connectivity on a large-scale. The equivalent of cluster-extent thresholding for graphs has been proposed, such as the Network Based Statistic [49], which estimates the probability of observing groups of linked, suprathreshold edges based on chance. However, inferences can only be made on groups of interconnected edges, not individual ones. In addition, there is a substantial loss of information in model-based approaches when conducting statistical inference on signals (functional connections) averaged over a group of subjects, and discounting the joint responses among many functional connections.

Here, we present a novel alternative to identify functional connections of interest based on their information content in machine-learning based multivariate pattern analyses that attempt to discriminate between two conditions that differ based on a parameter of interest (in this case the emotion expression of a presented face). For this we used linear filter feature selection and plotted classification accuracy vs. number of included features in order to determine the number of features required to distinguish between conditions, and then identified the top N features on neuroanatomical display.

### “Information content” of neural activity vs. neural interactivity

Large-scale functional connectivity and network analysis has been increasingly used as the tool of choice for extracting meaningful and understanding complex brain organization [17], [18]. In the current work we applied simple Pearson correlation to estimate the large-scale functional connectivity of implicit threat-related emotion and ambiguous facial processing using a block-design. Previous work based on simulations has indicated that correlation-based methods, including Pearson correlation, are in general quite successful in capturing true network connections [18]. Here we “validated” the estimated connections by testing whether a subset of features could be used to decode (“brain-read”) the emotional expression of the facial stimulus that was presented during each block. For this we applied Multivariate Pattern Analyses (MVPA) techniques similar to those used previously to decode categories of viewed stimuli [50]–[54], orientation [55], [56], and the decisions made during a near-threshold fearful face discrimination task [57].

In contrast to the above-mentioned studies, which applied MVPA to the activity of spatially distributed regions and/or voxels, in the current work we applied pattern analysis to the correlations, or *interactivity*, between regions distributed throughout the whole-brain. We compared the decoding accuracy when using correlations as features versus beta estimates, (i.e. summary measures of activation amplitudes for each condition for each voxel). We observed that the peak classification rate when using betas (76%, ∼1900 features) was significantly lower than that achieved using FC (100%, ∼25 features). Even with an additional, initial feature-selection based on the entire data set which positively biased results, peak decoding accuracies when using ∼4,000 beta values (92%) were lower than those reached when using only ∼25 correlations as features and unbiased feature selection (100%). This suggests that there is substantially more information, relevant to cognitive-emotional neural processing, that is contained in the interactions between regions than is typically realized through standard univariate approaches. However, it should be noted that this requires enough TRs (time-points) to compute meaningful correlations between brain regions for a particular condition, and would thus in general be impractical for decoding single-trial or event-related data.

We observed that using whole-brain, anatomically defined ROIs to define nodes for whole-brain FC estimation yielded much higher classification rates than using nodes that were functionally defined (either from other meta-analyses or coordinates defined from GLM analysis of these same data). This was not too surprising, as these functionally defined ROIs were smaller (6 mm radius spheres centered around peak F-value coordinates from the contrast of F>N obtained from the GLM vs. atlas-based masks), and hence provided considerably less coverage of the brain. In addition, the GLM framework relies on multiple assumptions (i.e. model/shape of hemodynamic response function, effects add linearly, etc.) [58] and regions that show activation to a stimulus (i.e. sustained increase in signal amplitude during the duration of a block) may not necessarily exhibit differential functional connectivity and vice versa. These observations further the notion that there exists substantial information in whole-brain large-scale functional connectivity patterns, the nodes of which may not be captured or revealed adequately through standard GLM approaches.

### Limitations

Previous simulations have raised concerns regarding the use of atlas-based approaches for parcellating the brain [18]. Because the spatial ROIs used to extract average time-series for a brain region do not likely match well the actual functional boundaries, BOLD time-series from neighboring nodes are likely mixed with each other. While this hampers the ability to detect true functional connections between neighboring regions, it has minimal effect on estimating functional connectivity between distant regions. This perhaps explains why in this study most of the functional connections that discriminated between fearful and neutral faces are long-distance. Future experiments using non-atlas based approaches would likely lead to better estimates of shorter-range functional connections. We also note that the current atlas-based approach may have under-sampled the prefrontal cortex, and that possible future improvements could break up the prefrontal regions into smaller pieces in order to sample more nodes from this area.

Using Pearson correlation, it is possible that any association between two brain regions is the result of a spurious association with a third brain region. Another limitation of the current study is the required amount of data used to extract quality features of brain activity. Our use of correlations as features required a substantial number of time points (i.e. 40 scans per condition per subject) relative to previous studies of decoding emotion perception. Given this, it was not feasible to sample enough examples within a single or few subjects as is typical in multivariate pattern analysis studies, and we instead pooled examples across multiple subjects. On the other hand, the fact that reliable classifiers could be learned using examples from separate subjects speaks to the generalizability of our obtained results.

## Supporting Information

Table S1 Node labels and MNI coordinates (spatial eigenmap peaks averaged over all subjects) used for whole-brain results presented in Figure 4 of main text.
(DOCX)
