# Supplementary material for: Decoding Unattended Fearful Faces with Whole-Brain Correlations: An Approach to Identify Condition-Dependent Large-Scale Functional Connectivity
Source: PLoS Comput Biol. 2012 Mar 29;8(3):e1002441. doi: 10.1371/journal.pcbi.1002441 (PMC3315448; doi:10.1371/journal.pcbi.1002441)
Supplement: Table S1 — Node labels and MNI coordinates (spatial eigenmap peaks averaged over all subjects) used for whole-brain results presented in Figure 4 of main text. (DOCX) [file pcbi.1002441.s001.docx]

Table S1

Decoding unattended fearful faces with whole-brain correlations: an approach to identify condition-dependent large-scale functional connectivity

Authors: Spiro P. Pantazatos^1,2,*^, Ardesheer Talati^3^, Paul Pavlidis^7,8^, Joy Hirsch^1,4,5,6,*^

^1^fMRI Research Lab, Depts of ^2^Physiology and Cellular Biophysics, ^3^Psychiatry, ^4^Neuroscience, ^5^Radiology, ^6^Psychology, Columbia University, New York, NY, USA; Dept of ^7^Psychiatry and ^8^Centre for High-throughout Biology, University of British Columbia, Vancouver, British Columbia, Canada

* To whom correspondence should be addressed:

E-mail: [spiropan@gmail.com](mailto:spiropan@gmail.com), joyhirsch@yahoo.com

**Table S1**

| **ROI** | **X** | **Y** | **Z** | **Name** |
| --- | --- | --- | --- | --- |
| 1 | -14 | -38 | -22 | Cerebelum_4_5_L_PC1 |
| 2 | -12 | -50 | -22 | Cerebelum_4_5_L_PC2 |
| 3 | 14 | -40 | -20 | Cerebelum_4_5_R_PC1 |
| 4 | 12 | -54 | -22 | Cerebelum_4_5_R_PC2 |
| 5 | -30 | -56 | -26 | Cerebelum_6_L_PC1 |
| 6 | -14 | -64 | -22 | Cerebelum_6_L_PC2 |
| 7 | 30 | -56 | -26 | Cerebelum_6_R_PC1 |
| 8 | 18 | -64 | -26 | Cerebelum_6_R_PC2 |
| 9 | -6 | -66 | -32 | Cerebelum_8_L_PC1 |
| 10 | -6 | -70 | -32 | Cerebelum_8_L_PC2 |
| 11 | 10 | -66 | -34 | Cerebelum_8_R_PC1 |
| 12 | 12 | -72 | -34 | Cerebelum_8_R_PC2 |
| 13 | -4 | -48 | -34 | Cerebelum_9_L_PC1 |
| 14 | -10 | -56 | -36 | Cerebelum_9_L_PC2 |
| 15 | 6 | -48 | -36 | Cerebelum_9_R_PC1 |
| 16 | 14 | -54 | -38 | Cerebelum_9_R_PC2 |
| 17 | -38 | -66 | -28 | Cerebelum_Crus1_L_PC1 |
| 18 | -16 | -74 | -30 | Cerebelum_Crus1_L_PC2 |
| 19 | 38 | -66 | -30 | Cerebelum_Crus1_R_PC1 |
| 20 | 20 | -74 | -32 | Cerebelum_Crus1_R_PC2 |
| 21 | -8 | -80 | -30 | Cerebelum_Crus2_L_PC1 |
| 22 | -6 | -72 | -32 | Cerebelum_Crus2_L_PC2 |
| 23 | 8 | -80 | -32 | Cerebelum_Crus2_R_PC1 |
| 24 | 16 | -82 | -34 | Cerebelum_Crus2_R_PC2 |
| 25 | 2 | 0 | -12 | Hypothalamus_PC1 |
| 26 | -4 | -6 | -8 | Hypothalamus_PC2 |
| 27 | -8 | 12 | -6 | Left_Accumbens_PC1 |
| 28 | -12 | 8 | -6 | Left_Accumbens_PC2 |
| 29 | -20 | -2 | -20 | Left_Amygdala_PC1 |
| 30 | -22 | -6 | -16 | Left_Amygdala_PC2 |
| 31 | -54 | -60 | 26 | Left_Angular_Gyrus_PC1 |
| 32 | -46 | -56 | 44 | Left_Angular_Gyrus_PC2 |
| 33 | -8 | 10 | 8 | Left_Caudate_PC1 |
| 34 | -12 | 0 | 20 | Left_Caudate_PC2 |
| 35 | -46 | -12 | 8 | Left_Central_Opercular_Cortex_PC1 |
| 36 | -50 | -16 | 16 | Left_Central_Opercular_Cortex_PC2 |
| 37 | 0 | 38 | 2 | Left_Cingulate_Gyrus_anterior_division_PC1 |
| 38 | 0 | 14 | 32 | Left_Cingulate_Gyrus_anterior_division_PC2 |
| **ROI** | **X** | **Y** | **Z** | **Name (continued)** |
| 39 | 0 | -48 | 28 | Left_Cingulate_Gyrus_posterior_division_PC1 |
| 40 | -4 | -50 | 2 | Left_Cingulate_Gyrus_posterior_division_PC2 |
| 41 | -2 | -82 | 30 | Left_Cuneal_Cortex_PC1 |
| 42 | 0 | -88 | 24 | Left_Cuneal_Cortex_PC2 |
| 43 | -2 | 64 | 22 | Left_Dorsal_Frontal_Pole_PC1 |
| 44 | -34 | 52 | 20 | Left_Dorsal_Frontal_Pole_PC2 |
| 45 | -34 | -68 | 52 | Left_Dorsal_Lateral_Occipital_Cortex_superior_division_PC1 |
| 46 | -12 | -82 | 48 | Left_Dorsal_Lateral_Occipital_Cortex_superior_division_PC2 |
| 47 | -2 | 52 | -12 | Left_Frontal_Medial_Cortex_PC1 |
| 48 | -6 | 42 | -12 | Left_Frontal_Medial_Cortex_PC2 |
| 49 | -42 | 18 | 0 | Left_Frontal_Operculum_Cortex_PC1 |
| 50 | -36 | 18 | 8 | Left_Frontal_Operculum_Cortex_PC2 |
| 51 | -38 | 16 | -20 | Left_Frontal_Orbital_Cortex_PC1 |
| 52 | -38 | 22 | -14 | Left_Frontal_Orbital_Cortex_PC2 |
| 53 | -44 | -18 | 6 | Left_Heschls_Gyrus_H1_and_H2_PC1 |
| 54 | -46 | -24 | 10 | Left_Heschls_Gyrus_H1_and_H2_PC2 |
| 55 | -18 | -20 | -20 | Left_Hippocampus_PC1 |
| 56 | -26 | -24 | -12 | Left_Hippocampus_PC2 |
| 57 | -54 | 14 | 10 | Left_Inferior_Frontal_Gyrus_pars_opercularis_PC1 |
| 58 | -52 | 16 | 20 | Left_Inferior_Frontal_Gyrus_pars_opercularis_PC2 |
| 59 | -50 | 30 | 10 | Left_Inferior_Frontal_Gyrus_pars_triangularis_PC1 |
| 60 | -50 | 28 | -4 | Left_Inferior_Frontal_Gyrus_pars_triangularis_PC2 |
| 61 | -50 | -40 | -20 | Left_Inferior_Temporal_Gyrus_posterior_division_PC1 |
| 62 | -48 | -36 | -18 | Left_Inferior_Temporal_Gyrus_posterior_division_PC2 |
| 63 | -50 | -56 | -18 | Left_Inferior_Temporal_Gyrus_temporooccipital_part_PC1 |
| 64 | -48 | -56 | -18 | Left_Inferior_Temporal_Gyrus_temporooccipital_part_PC2 |
| 65 | -44 | -2 | -4 | Left_Insular_Cortex_PC1 |
| 66 | -38 | 16 | 0 | Left_Insular_Cortex_PC2 |
| 67 | -2 | -70 | 10 | Left_Intracalcarine_Cortex_PC1 |
| 68 | -4 | -84 | 4 | Left_Intracalcarine_Cortex_PC2 |
| 69 | 0 | -2 | 54 | Left_Juxtapositional_Lobule_Cortex_Supp_Motor_cortex_PC1 |
| 70 | -2 | 0 | 64 | Left_Juxtapositional_Lobule_Cortex_Supp_Motor_cortex_PC2 |
| 71 | -46 | -74 | 2 | Left_Lateral_Occipital_Cortex_inferior_division_PC1 |
| 72 | -52 | -62 | 12 | Left_Lateral_Occipital_Cortex_inferior_division_PC2 |
| 73 | -10 | -56 | -2 | Left_Lingual_Gyrus_PC1 |
| 74 | -2 | -86 | -14 | Left_Lingual_Gyrus_PC2 |
| 75 | -48 | 16 | 40 | Left_Middle_Frontal_Gyrus_PC1 |
| 76 | -46 | 32 | 30 | Left_Middle_Frontal_Gyrus_PC2 |
| 77 | -48 | 0 | -24 | Left_Middle_Temporal_Gyrus_anterior_division_PC1 |
| **ROI** | **X** | **Y** | **Z** | **Name (continued)** |
| 78 | -56 | -2 | -22 | Left_Middle_Temporal_Gyrus_anterior_division_PC2 |
| 79 | -60 | -30 | -8 | Left_Middle_Temporal_Gyrus_posterior_division_PC1 |
| 80 | -62 | -26 | -10 | Left_Middle_Temporal_Gyrus_posterior_division_PC2 |
| 81 | -56 | -52 | 6 | Left_Middle_Temporal_Gyrus_temporooccipital_part_PC1 |
| 82 | -62 | -52 | -4 | Left_Middle_Temporal_Gyrus_temporooccipital_part_PC2 |
| 83 | -26 | -80 | -18 | Left_Occipital_Fusiform_Gyrus_PC1 |
| 84 | -24 | -70 | -10 | Left_Occipital_Fusiform_Gyrus_PC2 |
| 85 | 0 | -98 | 8 | Left_Occipital_Pole_PC1 |
| 86 | 0 | -96 | 16 | Left_Occipital_Pole_PC2 |
| 87 | -18 | -4 | -2 | Left_Pallidum_PC1 |
| 88 | -22 | -12 | -2 | Left_Pallidum_PC2 |
| 89 | -2 | 50 | 0 | Left_Paracingulate_Gyrus_PC1 |
| 90 | -2 | 34 | 34 | Left_Paracingulate_Gyrus_PC2 |
| 91 | -18 | -22 | -26 | Left_Parahippocampal_Gyrus_anterior_division_PC1 |
| 92 | -22 | -4 | -32 | Left_Parahippocampal_Gyrus_anterior_division_PC2 |
| 93 | -16 | -30 | -22 | Left_Parahippocampal_Gyrus_posterior_division_PC1 |
| 94 | -10 | -40 | -12 | Left_Parahippocampal_Gyrus_posterior_division_PC2 |
| 95 | -46 | -34 | 16 | Left_Parietal_Operculum_Cortex_PC1 |
| 96 | -54 | -30 | 22 | Left_Parietal_Operculum_Cortex_PC2 |
| 97 | -46 | -4 | -10 | Left_Planum_Polare_PC1 |
| 98 | -48 | -4 | 0 | Left_Planum_Polare_PC2 |
| 99 | -60 | -24 | 10 | Left_Planum_Temporale_PC1 |
| 100 | -60 | -32 | 16 | Left_Planum_Temporale_PC2 |
| 101 | -40 | -26 | 60 | Left_Postcentral_Gyrus_PC1 |
| 102 | -58 | -12 | 32 | Left_Postcentral_Gyrus_PC2 |
| 103 | -52 | 4 | 36 | Left_Precentral_Gyrus_PC1 |
| 104 | -42 | -14 | 62 | Left_Precentral_Gyrus_PC2 |
| 105 | 0 | -68 | 34 | Left_Precuneous_Cortex_PC1 |
| 106 | 0 | -66 | 64 | Left_Precuneous_Cortex_PC2 |
| 107 | -24 | 6 | 0 | Left_Putamen_PC1 |
| 108 | -28 | -8 | 0 | Left_Putamen_PC2 |
| 109 | -2 | 12 | -20 | Left_Subcallosal_Cortex_PC1 |
| 110 | -2 | 12 | -10 | Left_Subcallosal_Cortex_PC2 |
| 111 | -2 | 32 | 60 | Left_Superior_Frontal_Gyrus_PC1 |
| 112 | -24 | 10 | 64 | Left_Superior_Frontal_Gyrus_PC2 |
| 113 | -40 | -48 | 60 | Left_Superior_Parietal_Lobule_PC1 |
| 114 | -30 | -46 | 64 | Left_Superior_Parietal_Lobule_PC2 |
| 115 | -48 | -4 | -14 | Left_Superior_Temporal_Gyrus_anterior_division_PC1 |
| 116 | -52 | -2 | -8 | Left_Superior_Temporal_Gyrus_anterior_division_PC2 |
| **ROI** | **X** | **Y** | **Z** | **Name (continued)** |
| 117 | -62 | -24 | 4 | Left_Superior_Temporal_Gyrus_posterior_division_PC1 |
| 118 | -60 | -30 | 0 | Left_Superior_Temporal_Gyrus_posterior_division_PC2 |
| 119 | 0 | -74 | 14 | Left_Supracalcarine_Cortex_PC1 |
| 120 | -12 | -66 | 16 | Left_Supracalcarine_Cortex_PC2 |
| 121 | -60 | -34 | 36 | Left_Supramarginal_Gyrus_anterior_division_PC1 |
| 122 | -52 | -34 | 48 | Left_Supramarginal_Gyrus_anterior_division_PC2 |
| 123 | -62 | -48 | 24 | Left_Supramarginal_Gyrus_posterior_division_PC1 |
| 124 | -54 | -46 | 44 | Left_Supramarginal_Gyrus_posterior_division_PC2 |
| 125 | -34 | -6 | -34 | Left_Temporal_Fusiform_Cortex_anterior_division_PC1 |
| 126 | -38 | -10 | -28 | Left_Temporal_Fusiform_Cortex_anterior_division_PC2 |
| 127 | -28 | -38 | -22 | Left_Temporal_Fusiform_Cortex_posterior_division_PC1 |
| 128 | -38 | -26 | -22 | Left_Temporal_Fusiform_Cortex_posterior_division_PC2 |
| 129 | -34 | -58 | -22 | Left_Temporal_Occipital_Fusiform_Cortex_PC1 |
| 130 | -26 | -58 | -14 | Left_Temporal_Occipital_Fusiform_Cortex_PC2 |
| 131 | -40 | 8 | -26 | Left_Temporal_Pole_PC1 |
| 132 | -44 | 14 | -14 | Left_Temporal_Pole_PC2 |
| 133 | -4 | -30 | 6 | Left_Thalamus_PC1 |
| 134 | 0 | -20 | 12 | Left_Thalamus_PC2 |
| 135 | -44 | 46 | 2 | Left_Ventral_Frontal_Pole_PC1 |
| 136 | -2 | 62 | 4 | Left_Ventral_Frontal_Pole_PC2 |
| 137 | -48 | -70 | 28 | Left_Ventral_Lateral_Occipital_Cortex_superior_division_PC1 |
| 138 | -36 | -86 | 20 | Left_Ventral_Lateral_Occipital_Cortex_superior_division_PC2 |
| 139 | -8 | -34 | -16 | Midbrain_PC1 |
| 140 | 2 | -16 | -10 | Midbrain_PC2 |
| 141 | 16 | -30 | -28 | Pons_PC1 |
| 142 | -18 | -30 | -38 | Pons_PC2 |
| 143 | 8 | 12 | -6 | Right_Accumbens_PC1 |
| 144 | 10 | 16 | -8 | Right_Accumbens_PC2 |
| 145 | 18 | 0 | -20 | Right_Amygdala_PC1 |
| 146 | 24 | -6 | -16 | Right_Amygdala_PC2 |
| 147 | 58 | -54 | 28 | Right_Angular_Gyrus_PC1 |
| 148 | 56 | -54 | 28 | Right_Angular_Gyrus_PC2 |
| 149 | 10 | 12 | 8 | Right_Caudate_PC1 |
| 150 | 14 | 4 | 20 | Right_Caudate_PC2 |
| 151 | 48 | -8 | 6 | Right_Central_Opercular_Cortex_PC1 |
| 152 | 46 | -12 | 16 | Right_Central_Opercular_Cortex_PC2 |
| 153 | 0 | 36 | -2 | Right_Cingulate_Gyrus_anterior_division_PC1 |
| 154 | 2 | -8 | 34 | Right_Cingulate_Gyrus_anterior_division_PC2 |
| 155 | 2 | -40 | 32 | Right_Cingulate_Gyrus_posterior_division_PC1 |
| **ROI** | **X** | **Y** | **Z** | **Name (continued)** |
| 156 | 2 | -30 | 42 | Right_Cingulate_Gyrus_posterior_division_PC2 |
| 157 | 4 | -82 | 34 | Right_Cuneal_Cortex_PC1 |
| 158 | 2 | -70 | 24 | Right_Cuneal_Cortex_PC2 |
| 159 | 36 | 52 | 20 | Right_Dorsal_Frontal_Pole_PC1 |
| 160 | 2 | 64 | 22 | Right_Dorsal_Frontal_Pole_PC2 |
| 161 | 34 | -70 | 52 | Right_Dorsal_Lateral_Occipital_Cortex_superior_division_PC1 |
| 162 | 20 | -72 | 58 | Right_Dorsal_Lateral_Occipital_Cortex_superior_division_PC2 |
| 163 | 0 | 50 | -12 | Right_Frontal_Medial_Cortex_PC1 |
| 164 | 6 | 44 | -12 | Right_Frontal_Medial_Cortex_PC2 |
| 165 | 44 | 20 | 0 | Right_Frontal_Operculum_Cortex_PC1 |
| 166 | 38 | 18 | 8 | Right_Frontal_Operculum_Cortex_PC2 |
| 167 | 40 | 22 | -14 | Right_Frontal_Orbital_Cortex_PC1 |
| 168 | 16 | 6 | -20 | Right_Frontal_Orbital_Cortex_PC2 |
| 169 | 46 | -16 | 6 | Right_Heschls_Gyrus_H1_and_H2_PC1 |
| 170 | 46 | -20 | 12 | Right_Heschls_Gyrus_H1_and_H2_PC2 |
| 171 | 20 | -18 | -20 | Right_Hippocampus_PC1 |
| 172 | 26 | -24 | -12 | Right_Hippocampus_PC2 |
| 173 | 56 | 16 | 8 | Right_Inferior_Frontal_Gyrus_pars_opercularis_PC1 |
| 174 | 52 | 20 | 24 | Right_Inferior_Frontal_Gyrus_pars_opercularis_PC2 |
| 175 | 54 | 26 | 2 | Right_Inferior_Frontal_Gyrus_pars_triangularis_PC1 |
| 176 | 52 | 30 | 14 | Right_Inferior_Frontal_Gyrus_pars_triangularis_PC2 |
| 177 | 46 | 2 | -34 | Right_Inferior_Temporal_Gyrus_anterior_division_PC1 |
| 178 | 40 | 2 | -40 | Right_Inferior_Temporal_Gyrus_anterior_division_PC2 |
| 179 | 52 | -36 | -20 | Right_Inferior_Temporal_Gyrus_posterior_division_PC1 |
| 180 | 50 | -36 | -18 | Right_Inferior_Temporal_Gyrus_posterior_division_PC2 |
| 181 | 54 | -54 | -18 | Right_Inferior_Temporal_Gyrus_temporooccipital_part_PC1 |
| 182 | 58 | -46 | -16 | Right_Inferior_Temporal_Gyrus_temporooccipital_part_PC2 |
| 183 | 44 | 6 | -8 | Right_Insular_Cortex_PC1 |
| 184 | 42 | -6 | 8 | Right_Insular_Cortex_PC2 |
| 185 | 2 | -72 | 10 | Right_Intracalcarine_Cortex_PC1 |
| 186 | 8 | -84 | 6 | Right_Intracalcarine_Cortex_PC2 |
| 187 | 4 | 0 | 56 | Right_Juxtapositional_Lobule_Cortex_Supp_Motor_cortex_PC1 |
| 188 | 4 | -10 | 48 | Right_Juxtapositional_Lobule_Cortex_Supp_Motor_cortex_PC2 |
| 189 | 42 | -74 | -22 | Right_Lateral_Occipital_Cortex_inferior_division_PC1 |
| 190 | 46 | -74 | 6 | Right_Lateral_Occipital_Cortex_inferior_division_PC2 |
| 191 | 10 | -48 | -4 | Right_Lingual_Gyrus_PC1 |
| 192 | 4 | -80 | -12 | Right_Lingual_Gyrus_PC2 |
| 193 | 48 | 18 | 40 | Right_Middle_Frontal_Gyrus_PC1 |
| 194 | 34 | 34 | 46 | Right_Middle_Frontal_Gyrus_PC2 |
| **ROI** | **X** | **Y** | **Z** | **Name (continued)** |
| 195 | 48 | 2 | -24 | Right_Middle_Temporal_Gyrus_anterior_division_PC1 |
| 196 | 58 | -4 | -20 | Right_Middle_Temporal_Gyrus_anterior_division_PC2 |
| 197 | 62 | -20 | -12 | Right_Middle_Temporal_Gyrus_posterior_division_PC1 |
| 198 | 64 | -32 | -10 | Right_Middle_Temporal_Gyrus_posterior_division_PC2 |
| 199 | 60 | -48 | 4 | Right_Middle_Temporal_Gyrus_temporooccipital_part_PC1 |
| 200 | 56 | -54 | 0 | Right_Middle_Temporal_Gyrus_temporooccipital_part_PC2 |
| 201 | 30 | -74 | -20 | Right_Occipital_Fusiform_Gyrus_PC1 |
| 202 | 22 | -90 | -16 | Right_Occipital_Fusiform_Gyrus_PC2 |
| 203 | 6 | -100 | 4 | Right_Occipital_Pole_PC1 |
| 204 | 10 | -102 | 6 | Right_Occipital_Pole_PC2 |
| 205 | 20 | -2 | -2 | Right_Pallidum_PC1 |
| 206 | 24 | -10 | -2 | Right_Pallidum_PC2 |
| 207 | 0 | 50 | -2 | Right_Paracingulate_Gyrus_PC1 |
| 208 | 4 | 26 | 40 | Right_Paracingulate_Gyrus_PC2 |
| 209 | 18 | -22 | -26 | Right_Parahippocampal_Gyrus_anterior_division_PC1 |
| 210 | 8 | -4 | -26 | Right_Parahippocampal_Gyrus_anterior_division_PC2 |
| 211 | 18 | -28 | -22 | Right_Parahippocampal_Gyrus_posterior_division_PC1 |
| 212 | 16 | -34 | -16 | Right_Parahippocampal_Gyrus_posterior_division_PC2 |
| 213 | 56 | -28 | 18 | Right_Parietal_Operculum_Cortex_PC1 |
| 214 | 44 | -22 | 16 | Right_Parietal_Operculum_Cortex_PC2 |
| 215 | 48 | 0 | -10 | Right_Planum_Polare_PC1 |
| 216 | 50 | -6 | -2 | Right_Planum_Polare_PC2 |
| 217 | 60 | -24 | 12 | Right_Planum_Temporale_PC1 |
| 218 | 56 | -30 | 14 | Right_Planum_Temporale_PC2 |
| 219 | 58 | -14 | 40 | Right_Postcentral_Gyrus_PC1 |
| 220 | 52 | -22 | 52 | Right_Postcentral_Gyrus_PC2 |
| 221 | 54 | 6 | 34 | Right_Precentral_Gyrus_PC1 |
| 222 | 44 | -10 | 62 | Right_Precentral_Gyrus_PC2 |
| 223 | 2 | -76 | 44 | Right_Precuneous_Cortex_PC1 |
| 224 | 4 | -80 | 50 | Right_Precuneous_Cortex_PC2 |
| 225 | 26 | 6 | 0 | Right_Putamen_PC1 |
| 226 | 30 | -6 | 4 | Right_Putamen_PC2 |
| 227 | 2 | 10 | -18 | Right_Subcallosal_Cortex_PC1 |
| 228 | 2 | 14 | -12 | Right_Subcallosal_Cortex_PC2 |
| 229 | 2 | 32 | 60 | Right_Superior_Frontal_Gyrus_PC1 |
| 230 | 24 | 10 | 64 | Right_Superior_Frontal_Gyrus_PC2 |
| 231 | 38 | -48 | 60 | Right_Superior_Parietal_Lobule_PC1 |
| 232 | 32 | -44 | 54 | Right_Superior_Parietal_Lobule_PC2 |
| 233 | 60 | 4 | -8 | Right_Superior_Temporal_Gyrus_anterior_division_PC1 |
| **ROI** | **X** | **Y** | **Z** | **Name (continued)** |
| 234 | 56 | -2 | -12 | Right_Superior_Temporal_Gyrus_anterior_division_PC2 |
| 235 | 64 | -16 | 2 | Right_Superior_Temporal_Gyrus_posterior_division_PC1 |
| 236 | 62 | -12 | -8 | Right_Superior_Temporal_Gyrus_posterior_division_PC2 |
| 237 | 2 | -76 | 14 | Right_Supracalcarine_Cortex_PC1 |
| 238 | 18 | -64 | 16 | Right_Supracalcarine_Cortex_PC2 |
| 239 | 64 | -28 | 32 | Right_Supramarginal_Gyrus_anterior_division_PC1 |
| 240 | 56 | -30 | 48 | Right_Supramarginal_Gyrus_anterior_division_PC2 |
| 241 | 62 | -40 | 38 | Right_Supramarginal_Gyrus_posterior_division_PC1 |
| 242 | 64 | -44 | 18 | Right_Supramarginal_Gyrus_posterior_division_PC2 |
| 243 | 34 | -6 | -38 | Right_Temporal_Fusiform_Cortex_anterior_division_PC1 |
| 244 | 40 | -8 | -32 | Right_Temporal_Fusiform_Cortex_anterior_division_PC2 |
| 245 | 30 | -34 | -24 | Right_Temporal_Fusiform_Cortex_posterior_division_PC1 |
| 246 | 38 | -28 | -22 | Right_Temporal_Fusiform_Cortex_posterior_division_PC2 |
| 247 | 26 | -48 | -18 | Right_Temporal_Occipital_Fusiform_Cortex_PC1 |
| 248 | 42 | -52 | -24 | Right_Temporal_Occipital_Fusiform_Cortex_PC2 |
| 249 | 40 | 12 | -26 | Right_Temporal_Pole_PC1 |
| 250 | 46 | 10 | -12 | Right_Temporal_Pole_PC2 |
| 251 | 6 | -30 | 6 | Right_Thalamus_PC1 |
| 252 | 2 | -8 | 0 | Right_Thalamus_PC2 |
| 253 | 38 | 52 | -8 | Right_Ventral_Frontal_Pole_PC1 |
| 254 | 0 | 62 | 4 | Right_Ventral_Frontal_Pole_PC2 |
| 255 | 50 | -68 | 28 | Right_Ventral_Lateral_Occipital_Cortex_superior_division_PC1 |
| 256 | 32 | -84 | 28 | Right_Ventral_Lateral_Occipital_Cortex_superior_division_PC2 |
| 257 | 2 | -46 | -30 | Vermis_10_PC1 |
| 258 | 0 | -52 | -26 | Vermis_10_PC2 |
| 259 | 2 | -44 | -14 | Vermis_3_PC1 |
| 260 | 6 | -46 | -14 | Vermis_3_PC2 |
| 261 | 0 | -46 | -8 | Vermis_4_5_PC1 |
| 262 | 2 | -52 | -18 | Vermis_4_5_PC2 |
| 263 | 2 | -66 | -20 | Vermis_6_PC1 |
| 264 | 2 | -60 | -24 | Vermis_6_PC2 |
| 265 | 2 | -74 | -28 | Vermis_7_PC1 |
| 266 | 2 | -64 | -26 | Vermis_7_PC2 |
| 267 | 2 | -62 | -32 | Vermis_8_PC1 |
| 268 | 0 | -68 | -34 | Vermis_8_PC2 |
| 269 | 2 | -54 | -34 | Vermis_9_PC1 |
| 270 | 8 | -58 | -36 | Vermis_9_PC2 |
